# How Advanced Is Our Understanding of the Role of Intestinal Barrier Dysfunction in the Pathogenesis of Recurrent Urinary Tract Infections

**DOI:** 10.3389/fphar.2022.780122

**Published:** 2022-03-10

**Authors:** Natalia Stepanova

**Affiliations:** State Institution “Institute of Nephrology National Academy of Medical Science of Ukraine”, Kyiv, Ukraine

**Keywords:** recurrent urinary tract infections, intestinal barrier dysfunction, dysbiosis, enteric inflammation, uropathogen

## Abstract

A comprehensive understanding of urinary tract infections (UTIs), one of the most common human infections, is required as they are complex and poorly understood diseases. Periurethral and vaginal colonization by rectal flora, with the constant presence of pathogens in the urethra, is the initial step of the recurrent UTIs pathway. Current scientific data describe the genetic, etiological, biological, and behavioral risk factors for recurring UTIs, but they do not include the effect of intestinal barrier function on the disease. Although gut microbiota has been proposed as the main source for UTIs, the cross-talk between intestinal barrier dysfunction and the recurrence of UTIs has not yet been supported by scientific data. In this opinion review, based on published data and the results of our clinical studies, I aimed to outline the possible contribution of intestinal barrier dysfunction to the pathogenesis of recurrent UTIs. I believe that the unanswered questions raised by this review can guide further experimental and controlled studies to clarify the mechanisms underlying the role of intestinal barrier dysfunction in the pathogenesis of recurrent UTIs.

## Introduction

Urinary tract infections (UTIs) are a worldwide weighty medical, economic, and social burden due to their high prevalence among sexually active women (Kolesnyk et al., 2014; Kolesnyk et al., 2016; Gaĭ;seniuk et al., 2013; Kolman, 2019). About 70% of sexually active premenopausal women have at least one episode of UTIs in their lives, while 25–44% have recurrent forms of UTIs (i.e., two episodes per 6 months or three or more recurrences during a year) (Foxman, 2014; Ke et al., 2021). Recurrent UTIs (RUTIs) are associated with significant financial expenses for both patients and government because of the cost of diagnostic tests and antibacterial therapy (Glover et al., 2014; Magruder et al., 2019). The emergence of multidrug-resistant bacterial strains and, more importantly, unsatisfactory treatment outcomes for both patients and physicians necessitate the search for alternative and advanced medical solutions.

The normal functioning of the gastrointestinal tract is balanced between the microbiota composition and the maintenance of the permeability of the mucosal barrier (Turner, 2009; Meijers et al., 2018; Ghosh et al., 2021). Under physiological conditions, the intestinal barrier is both permeable to nutrients and macromolecules and serves as a protective shield against pathogens and harmful substances (Turner, 2009; Ghosh et al., 2021). An imbalance in either the microbiota composition or the structural components of the intestinal barrier can result in increased intestinal permeability, leading to bacterial translocation and inflammation (Turner, 2009; Vancamelbeke and Vermeire, 2017; Muehler et al., 2020; Ghosh et al., 2021). However, the view of the intestinal barrier as simply a physical barrier that separates our body from the external environment has lost its relevance. In addition to the gut microbiome, many elements, such as short-chain fatty acids, tight junction proteins, mucins, secretory immunoglobulin A (sIg A), antimicrobial peptides, enzymes, and various other cellular regulators, play crucial roles in appropriate intestinal barrier functional integrity (Nagpal and Yadav, 2017; Vancamelbeke and Vermeire, 2017). Moreover, cytokines and chemokines, secreted by epithelial cells and stimulated by the gut microbiota, modulate host immune responses, which maintain the host’s immune system (Okumura and Takeda, 2017; Vancamelbeke and Vermeire, 2017). Accordingly, disruption of the composition and functioning of the microbiota and/or the intestinal barrier may be associated not only with intestinal inflammation but also inflammatory bowel diseases. Current knowledge of the relevance of the intestinal barrier’s function is providing increasing evidence of its interaction with neurological and autoimmune disease, non-alcoholic fatty liver disease, obesity and diabetes, rheumatoid arthritis, and chronic kidney diseases (Turner, 2009; Okumura and Takeda, 2017; Vancamelbeke and Vermeire, 2017; Meijers et al., 2018; Barbosa and Barbosa, 2020; Muehler et al., 2020). However, although antibiotics remain the basis of RUTIs treatment and prevention, there is a general lack of evidence for altered intestinal barrier function in patients with RUTIs. In this opinion review, I summarized the latest scientific data to outline the possible role of intestinal barrier dysfunction in the pathogenesis of RUTIs.

### Routes of Urinary Tract Infection: An Old Issue With New Questions

Currently, two main routes of uropathogenic invasion are considered: an ascending route, which is realized through bacterial entry to the urothelium from colonies in the periurethral area, vagina, and/or rectum, and a hematogenous route (Stapleton, 2016; Meštrović et al., 2020). In physiological conditions, the glycosaminoglycan layers of urothelial plaques protect against bacterial colonization through urothelial cells and prevent microbial penetration (Abraham and Miao, 2015; Dalghi et al., 2020). The presence of any risk or complicating factor (e.g., female sex, UTI history, anatomical or functional abnormalities of the urinary tract, obstruction, vesicoureteral reflux, urine catheterization, and pregnancy or post-menopausal age) compromises the urothelial barrier function, that in response to uropathogenic *Escherichia coli* (UPEC) entry, leads to host inflammatory responses (Hooton, 2012; Abraham and Miao, 2015; Meštrović et al., 2020). However, most cases of uncomplicated UTIs cannot be explained by the presence of anatomical abnormalities or functional disorders. Therefore, obvious questions arise: *Why doesn’t everyone develop UTIs under the same conditions?* and *Why do recurrences develop in the absence of complicating factors?* However, unambiguous answers to these and many other questions are yet to be found. The value of genetic, hormonal, and/or metabolic factors; emotional and behavioral influences; the effect of urine or vaginal pH; inadequate local antibody production; and numerous other factors in the pathogenesis of uncomplicated UTIs have been actively discussed (Hooton, 2012; Foxman, 2014; Flores-Mireles et al., 2015; Behzadi, 2019; Storme et al., 2019; Wagenlehner et al., 2020). Moreover, the ability of UPEC to form virulence factors (e.g., adhesins, capsules, siderophores, and toxins) is a crucial facilitator of bacterial colonization and urothelium damage resulting in inflammation (Abraham and Miao, 2015; Terlizzi et al., 2017; Bunduki et al., 2021).

Nevertheless, despite the absence of exact answers to these questions, the ascending route of UTIs seems to be the most convincing, while the possibility of the hematogenous route of UTIs, in particular pyelonephritis, is a “standard” statement, which is yet to be confirmed in the absence of complicating factors. On the one hand, the hematogenous route is logical because the kidneys receive 20–25% of cardiac output (Kaufman et al., 2021), and any microorganism can be delivered to them. On the other hand, the same conditions of blood supply must provide sufficient resistance to the renal parenchyma and, therefore, the necessity of other conditions for pyelonephritis development to become obvious. Several early experimental studies have proven the hematogenous spread of UPEC to the kidneys (Larsson et al., 1980; Tanaka et al., 1981). However, no one has shown the possibility of pyelonephritis as a consequence of the bloodstream dissemination of Gram-negative bacteria without temporary occlusion of the ureter (Tancheva et al., 2011; Sullivan and Ulett, 2020). It should be emphasized that, unlike Gram-negative bacteria, group B *streptococcus* bacteria or *Candida spp*. can be disseminated to the kidneys via the hematogenous route even with a single injection of an appropriate volume and number of virulent bacteria (Sullivan and Ulett, 2020). These data can explain pyelonephritis in patients with septicemia caused by Gram-positive bacteria and negative urine cultures but not the development of RUTIs. In this context, the only explanation for uncomplicated RUTIs is an ascending route via intestinal, vaginal, or urinary dysbiosis. Nonetheless, *should we always assume the obvious as true?*

### Altered Gut Microbiota: A Source or Consequence of Recurrent Urinary Tract Infections

The gut microbiota has been proposed as the main source of UTIs (Flores-Mireles et al., 2015; Stapleton, 2016; Magruder et al., 2019; Meštrović et al., 2020). Indeed, the intestinal origin of UPEC in patients with UTIs is supported by numerous studies (Paalanne et al., 2018; Magruder et al., 2019, 2020; Klein and Hultgren, 2020; Meštrović et al., 2020). For example, recent studies by Magruder et al. have demonstrated that an increased gut abundance of Enterobacteriaceae is associated with bacteriuria and the future development of UTIs in kidney transplant recipients (Magruder et al., 2019, 2020). Forde et al., after observing the dynamics of *Escherichia coli* (*E. coli*) strain 131 over 5 years in a woman with RUTIs, indicated the intestine as a reservoir for the recurrences (Forde et al., 2019). Moreover, current progress in metagenomic approaches has overturned the existing medical dogma of urinary tract sterility and demonstrated a large diversity of microbial species in the urine of patients without any clinical symptoms (Lewis et al., 2013; Neugent et al., 2020; Price et al., 2020). In addition, recent data provided by Thomas-White et al. (Thomas-White et al., 2018) and Price et al. (Price et al., 2020) have evidenced that not only UPEC but other different commensals, such as *Lactobacillus spp*., can be identified in the urinary bladder microbiota, suggesting a gut-derived source of pathogens. These new findings have thrown up many questions in need of further investigation: *Does the gut microbiome cause the diversity of the urine microbiome? Does the urine microbiome change over time? Can we change the diversity of urinary commensals by influencing the gut microbiome?*

UPEC are a pathotype of extraintestinal pathogenic *E. coli* (ExPEC) that can adapt to the competitive and volatile intestinal environment and colonize the urinary tract due to their numerous virulence factors and adhesive ability (Mann et al., 2017; Sarowska et al., 2019). The virulence factors of UPEC can be roughly divided into two groups: the bacterial cell surface factors (a number of different types of fimbriae, flagellum, capsular lipopolysaccharide, outer membrane proteins) and the secreted virulence factors (α-hemolysin, cytotoxic necrotizing factor 1, siderophores) (Behzadi, 2019; Sarowska et al., 2019; Shah et al., 2019; Khonsari et al., 2021). Both type 1 and P fimbriae encoded by *fim* and *pap* operon genes, respectively, are among the best-characterized bacterial fimbriae that promote adhesion to host cell surfaces, tissue invasion, biofilm formation, and cytokine expression (Terlizzi et al., 2017; Behzadi, 2019; Shah et al., 2019; Khonsari et al., 2021). Apart from adhesins, hemolysin, cytotoxic-necrotizing-factor, and siderophores encoded by *hlyA*, *cnf1*, and *fyuA* genes, respectively, are involved in intracellular survival, iron acquisition, host immune response, and tissue damage (Behzadi, 2019; Shah et al., 2019; Hozzari et al., 2020). To put the mentioned simply, UPEC possesses a huge number of virulent genes and factors, which allows them to develop different types of UTIs. It has been demonstrated that the phylogenetic group status which is based on the genomic Pathogenicity Islands and their associated virulence genes may contribute to the development of RUTIs and correlate with antibiotic resistance patterns (Luo et al., 2012; Shah et al., 2019; Hozzari et al., 2020). The phylogenetic groups B2 and D are traditionally considered to be the most virulent strains of UPEC (Behzadi, 2019; Khonsari et al., 2021) but recently, high percentages of phylogroup A strains have also been identified in UTI cases (Khairy et al., 2019). Nevertheless, despite the fundamental role of virulence factors in UPEC colonization and persistence, host epithelial dysfunction and its immune response are an integral part of UTI causing (Hooton, 2012; Sarowska et al., 2019; Meštrović et al., 2020).

In healthy individuals, ExPEC ingested with contaminated food or water are destroyed by the gut’s microbial community, while the intestinal epithelial layer creates a barrier against bacterial invasion (Mann et al., 2017; Govindarajan et al., 2020). Host epithelium dysfunction leads to bacterial imbalance and the overabundance of ExPEC in the intestine, resulting in the overproduction of toxins, local inflammation, and dysbiosis (Mann et al., 2017; Govindarajan et al., 2020). Additionally, ExPEC has been shown to specifically interact with the intestinal epithelial barrier (Bischoff et al., 2014; Francino, 2016; Poole et al., 2017) and trigger intestinal barrier dysfunction before the onset of disease (Turner, 2009; Nagpal and Yadav, 2017). The best example of this interaction is the regulation of epithelial barrier function by the activation of Toll-like receptors (TLRs). The main function of TLRs is the rapid recognition of pathogens (e.g., bacteria, viruses, and fungi) and the signaling of their unauthorized penetration of anatomical barriers (Vijay, 2018; El-Zayat et al., 2019; Behzadi et al., 2021). TLR signals regulate the activation of innate immunity and provide a relationship with acquired immunity through antigen-presenting cells (APCs) (Behzadi and Behzadi, 2016; Vijay, 2018; El-Zayat et al., 2019; Behzadi et al., 2021; Ghosh et al., 2021). Once the pathogenic bacteria adhere to the epithelium, TLRs on the surface of the APCs bind to components of the infected cells, such as the lipopolysaccharides (LPSs) of Gram-negative bacteria, and induce APCs to produce cytokines (Behzadi and Behzadi, 2016; Ghosh et al., 2021). In turn, LPSs disrupt junctional protein complexes and activate inflammatory cytokines released by TLR signaling, leading to increased gut permeability and bacterial translocation (Behzadi and Behzadi, 2016; Vijay, 2018; El-Zayat et al., 2019; Ghosh et al., 2021).

However, besides the overabundance of ExPEC *per se,* antibiotics can violate the quantitative and qualitative composition of gut microbiota and promote dysbiosis and the intestinal colonization of antibiotic-resistant bacteria (Lau et al., 2015; Yang et al., 2018; Stepanova, 2021). Patients with RUTIs must constantly take antibiotics, including long-term antibiotic prophylaxis (Fine et al., 2019; Issakhanian and Behzadi, 2019; Toor et al., 2019; Meštrović et al., 2020). Although antibiotics prevent UTI recurrences, antibiotic-induced dysbiosis leads to increased production and accumulation of LPSs and other toxic products of bacterial activity (e.g., P-cresol, trimethylamine-N-oxidoreductase, and indoxyl sulfate) and intestinal epithelial barrier dysfunction that accelerates excessive production of cytokines and chemokines and facilitates their translocation, which causes extraintestinal and/or systemic inflammation (Driyanska et al., 2014; Francino, 2016; Stepanova et al., 2018a; Gagliardi et al., 2018). It should be noted that the effect of antibiotics on intestinal barrier function is still poorly understood. In a recent *in vivo* study, Feng et al. examined the mechanisms of the effect of antibiotics on intestinal barrier function and suggested they induce tight junction dysfunction via decreasing expression of their proteins and the alteration zonula occludens protein 1 morphology (Feng et al., 2019). Moreover, the authors demonstrated antibiotic-derived activation of NLRP3 inflammasome and autophagy, which has been reported to be associated with intestinal barrier dysfunction (Feng et al., 2019). Holota et al. in a rat model showed an increase in cecum weight and a decrease in the level of mucus glycoproteins with a change in their carbohydrate composition in 56 days after antibiotic withdrawal (Holota et al., 2019). They also found changes in matrix metalloproteinase-2 and -9 activity, morphological remodeling of colon tissue, and increased colonic epithelial permeability to Evans blue dye, which was further confirmed by increased bacterial translocation from the lumen to the blood as late as 56 days after the antibiotic withdrawal (Holota et al., 2019). These experimental data provide evidence of long-term antibiotic-induced impairment of intestinal permeability that facilitates the translocation of pathogenic bacteria, antigens, and other microbial products to the systemic circulation leading to chronic inflammation (Johnson et al., 2015; Poole et al., 2017; Sharapatov et al., 2021). Gut microbiota-derived inflammation, so-called metabolic endotoxemia, has been recently demonstrated to play a key role in the pathogenesis of many diseases such as obesity, type 2 diabetes, pancreatitis, cardiovascular, systemic, kidney, liver, and bone diseases, as well as brain and mental disorders (Durack and Lynch, 2019; Barbosa and Barbosa, 2020; Ilchmann-Diounou and Menard, 2020; Ren et al., 2020; Skinner et al., 2020; Zhou et al., 2020). Interestingly, endotoxemia and low-level inflammation caused by increased intestinal permeability have also been demonstrated in psychological stress and depression, which often coexist with RUTIs (de Punder and Pruimboom, 2015; Trzeciak and Herbet, 2021). Given that after entering the bloodstream, pathogens and toxins can be delivered within one to 2 minutes to the vascular bed in any part of the body (Nash et al., 2015), *why do we consider such a possibility for the above diseases and exclude the same for RUTIs?*

Unfortunately, there are no evidenced clinical data on bacterial translocation in uncomplicated UTIs partially because of the methodological limitations. However, in a recent *in vitro* study, Owrangi et al. have demonstrated that UPEC strains isolated from patients with community-acquired UTIs not only had a similar rate of translocation through culturing human gut epithelium Caco-2 cells, but also translocated significantly more compared to UPEC strains from patients with urosepsis. Nonetheless, the virulence potential of urosepsis UPEC strains was significantly greater compared to community-acquired UTI strains, which allowed them to cause blood infection (Owrangi et al., 2018). Again, bacterial translocation is a constant physiological process that at a low level can be observed in healthy people and does not always manifest clinically. Most of the patients with bacterial translocation to mesenteric lymph nodes (the most reliable method of bacterial translocation evaluating in humans) showed no clinical infectious complications (Vaishnavi, 2013; Mohammad and Thiemermann, 2021). Besides, similar to the urinary microbiome, recent advances in culture-independent methods rejected the dogma of blood “sterility” and the existence of a “healthy” human blood microbiome has been demonstrated (D’Aquila et al., 2021; Castillo et al., 2019; Shah N. B et al., 2019). Unlike the clinical relevance of gut dysbiosis to human health, the problem of blood microbiome dysbiosis has only been addressed in a limited number of studies. However, the published data have indicated the changes of blood microbial taxonomic diversity in different diseases (Potgieter et al., 2015; Castillo et al., 2019; Shah N. B et al., 2019). Therefore, it is quite possible that the clinical relevance of bacterial translocation, and the “dormant” blood microbiome in patients with RUTIs, could depend on immune response and bacterial virulence properties influencing the severity and frequency of the infection.

Together, in my opinion, both the composition of the vaginal and urinary microbiota, as a consequence of gut dysbiosis, and the possible impact of the intestinal barrier dysfunction on bacterial translocation and chronic low-grade inflammation can trigger recurrences of uncomplicated UTI.

### Is Intestinal Barrier Dysfunction Clinically Significant in Recurrent Urinary Tract Infections

Since 1885, when Theodor Escherich first identified *E. coli* (Shulman et al., 2007), this pathogen has remained the postulated dominant causative agent of UTIs. However, currently, Gram-positive bacteria (i.e., *Enterococcus* spp.*, Streptococcus* spp.*, Staphylococcus saprophyticus, Staphylococcus epidermidis*, and *S. aureus*) have confidently filled the niche of UTIs pathogens (Kline and Lewis, 2016; Kolesnyk et al., 2016; Gajdács et al., 2020; Petca et al., 2020). As stated in various research, the identification of Gram-positive bacteria in outpatients with UTIs rose steadily from 2% in 1971, up to 5.6% in 1990 (Felmingham et al., 1992), and up to 26% between 2008 and 2017 (Gajdács et al., 2020). According to our data, the frequency of Gram-positive bacteria identification depends on the recurrence rate, and it accounts for 40% of cases of uncomplicated RUTIs (Kolesnyk et al., 2016). However, compared to *E. coli*, Gram-positive bacteria are more often shed in complicated UTIs and associated with catheters, functional or anatomical abnormalities, comorbidities, immunosuppression, and nosocomial infections (Kline and Lewis, 2016; Petca et al., 2020). Therefore, the following question arises: *How can we explain the significant spread of Gram-positive bacteria in uncomplicated RUTIs?*

**FIGURE 1:**
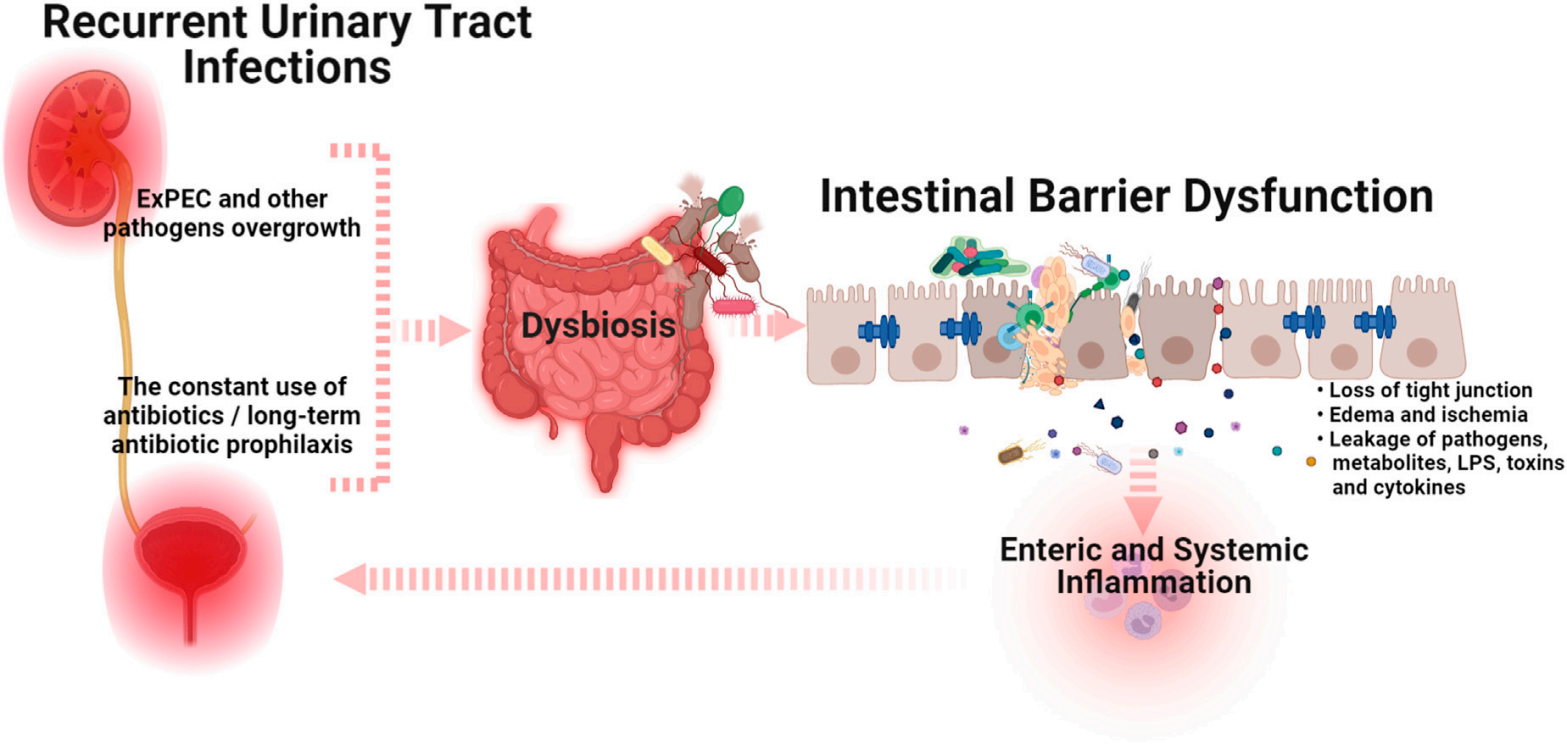
A schematic illustration of the cross-talk between intestinal barrier dysfunction and recurrent urinary tract infections (Graphic created by BioRender.com). The overgrowth of extraintestinal pathogenic *E. coli* (ExPEC) and/or the use of antibiotics that induce dysbiosis can trigger intestinal barrier dysfunction, which causes enteric and systemic inflammation. In turn, the impaired intestinal barrier is the main source of the constant persistence of ExPEC in the urinary tract and a consequent predisposition for RUTIs. Abbreviations: ExPEC, extraintestinal pathogenic *E. coli*; LPS, lipopolysaccharides.

Again, the use of broad-spectrum antibiotics resulting in intestinal barrier dysfunction is the obvious explanation. However, unfortunately, in the current era of microbiome research, this issue has not been supported by scientific data. Based on comprehensive clinical examinations, we have previously demonstrated that all women with uncomplicated RUTIs were diagnosed with gut dysbiosis and decreased contents of *Lactobacillus spp*. and *Bifidobacteria,* and an increased number of conditionally pathogenic enterobacteria, *E. coli*, *Clostridium*, *and Staphylococcus spp*. in feces samples (Stepanova et al., 2017; Stepanova et al., 2018b). It should be noted, that all the examined patients had increased levels of sIg A and myeloperoxidase activity in coprofiltrates, which suggested enteric inflammation, while only 20% had clinical symptoms of gut dysbiosis. These data have been recently confirmed in a year-long study by Worby et al. in which the authors showed strong similarities between gut dysbiosis in RUTIs and inflammatory bowel disease characterized by depleted levels of butyrate-producing bacteria and diminished microbial richness (Worby et al., 2021). Moreover, the authors found a higher plasma marker of intestinal inflammation, eotaxin-1, in women with RUTIs compared to healthy controls, suggesting low-level enteric inflammation (Worby et al., 2021). The presence of such subclinical enteric inflammation in patients with RUTIs indicates the formation of a vicious circle. The overgrowth of ExPEC or antibiotic-induced intestinal barrier dysfunction causes enteric and systemic inflammation. In turn, the impaired intestinal barrier is the main source of the constant persistence of ExPEC in the urinary tract and a consequent predisposition for RUTIs (Figure 1). This leads to the next question: *Should intestinal barrier dysfunction be considered a complicating factor for RUTIs?* If yes, understanding the approaches that can improve intestinal barrier function may unlock new therapeutic strategies for the treatment and prevention of RUTIs. In addition, an honest answer to the question, *How often do we prescribe an additional examination to exclude intestinal dysbiosis or barrier dysfunction in women without clinical manifestations?* could significantly reduce the prevalence of uncomplicated RUTIs. In our experience, in the absence of clinical symptoms, the diagnosis of gut dysbiosis and/or intestinal barrier dysfunction is usually not carried out and remains unrecognized. I in no way urge readers to prescribe unreasonable, numerous, and expensive diagnostic methods, and do not underestimate the significance of other complicating factors for RUTIs. I simply call on readers to think about the diagnostic necessity and significance of intestinal barrier dysfunction in the pathogenesis of RUTIs.

## Conclusion and Perspectives

Multiple environmental, genetic, host-related, and virulence risk factors play a determining role in the development of uncomplicated RUTIs. However, whatever the trigger, impaired intestinal barrier function appears to play a central role in urinary tract, enteric, and/or systemic inflammation. Although cross-talk between intestinal barrier dysfunction and RUTIs is not yet thoroughly understood, in this opinion review, I have presented a scientific rationale and underlined the possible role of intestinal barrier dysfunction in the pathogenesis of RUTIs. I believe that addressing the questions posed and many other questions will fill the knowledge gap on how intestinal barrier dysfunction is caused and how it can be restored in patients with RUTIs. As more and more research focuses on intestinal barrier function and its modulation, further potential therapeutic targets will result in the expansion of existing strategies for the treatment and prevention of RUTIs and provide new options for minimizing the prescription of antibiotics.
